# Bistable Perception Is Biased by Search Items but Not by Search Priming

**DOI:** 10.1177/2041669518812485

**Published:** 2018-12-11

**Authors:** M. A. B. Brinkhuis, J. W. Brascamp, Á. Kristjánsson

**Affiliations:** Department of Psychology, University of Iceland, Reykjavík, Iceland; Department of Psychology, Michigan State University, East Lansing, MI, USA; Department of Psychology, University of Iceland, Reykjavík, Iceland

**Keywords:** attention, perception, perceptual organization, visual memory, visual search

## Abstract

During visual search, selecting a target facilitates search for similar targets in the future, known as search priming. During bistable perception, in turn, perceiving one interpretation facilitates perception of the same interpretation in the future, a form of sensory memory. Previously, we investigated the relation between these history effects by asking: can visual search influence perception of a subsequent ambiguous display and can perception of an ambiguous display influence subsequent visual search? We found no evidence for such influences, however. Here, we investigated one potential factor that might have prevented such influences from arising: lack of retinal overlap between the ambiguous stimulus and the search array items. In the present work, we therefore interleaved presentations of an ambiguous stimulus with search trials in which the target or distractor occupied the same retinal location as the ambiguous stimulus. Nevertheless, we again found no evidence for influences of visual search on bistable perception, thus demonstrating no close relation between search priming and sensory memory. We did, however, find that visual search items primed perception of a subsequent ambiguous stimulus at the same retinal location, regardless of whether they were a target or a distractor item: a form of perceptual priming. Interestingly, the strengths of search priming and this perceptual priming were correlated on a trial-to-trial basis, suggesting that a common underlying factor influences both.

## Introduction

The human visual system represents the physical world to guide behavior in a useful way. This representation, however, can only be an approximation of the environment, due to physiological restrictions as well as inconclusive sensory information. Consequently, the meaning of a visual scene becomes ambiguous in certain conditions. Typically, in such a situation, the visual system prefers one interpretation over the other (Pastukhov et al., 2013). Experimentally, this can manifest as bistable perception of ambiguous figures—meaning that an observer sees different interpretations of the same ambiguous stimulus in alternation. Many studies have shown that, when such stimuli are shown repeatedly, a type of sensory memory due to previous presentations biases perception at the start of a subsequent presentation (de Jong, Knapen, & van Ee, 2012; Leopold, Wilke, Maier, & Logothetis, 2002; Maier, Wilke, Logothetis, & Leopold, 2003; Pastukhov, 2016; Pastukhov & Braun, 2008; Pearson & Brascamp, 2008).

We have previously suggested that this dependency on prior history could be related to priming in visual search (Brinkhuis, Kristjánsson, & Brascamp, 2015). In particular, when the features of a target, such as its shape or color, repeat between trials, search response times (RTs) decrease (Maljkovic & Nakayama, 1994; Treisman & Gelade, 1980) and the number of correct responses increases (Ásgeirsson, Kristjánsson, & Bundesen, 2014; Sigurdardottir, Kristjánsson, & Driver, 2008). The same is true when distractor features repeat between trials (Chetverikov, Campana, & Kristjánsson, 2016; Kristjánsson & Driver, 2008; Lamy, Yashar, & Ruderman, 2013; Tipper, 1985), whereas when target and distractor features are reversed, performance goes down (Kristjánsson & Driver, 2008). We considered that this target versus distractor bias may be analogous to a perceptual bias that is elicited by the current dominant percept of an ambiguous stimulus. In the previous study, we examined potential links between these two kinds of history effects, by presenting a search priming paradigm where search displays were interleaved with ambiguous displays. To investigate potential interactions, the target and distractors resembled the two perceptual interpretations of the ambiguous stimulus. We confirmed that visual search elicited search priming, and that bistable perception elicited sensory memory, but found no influence of either kind of trial on the other: Prior search trials did not bias subsequent bistable perception, and prior ambiguous stimuli did not affect subsequent visual search. This suggested that the two kinds of history-dependence, search priming and sensory memory, are unrelated.

The present work constitutes a closer examination of this earlier result, motivated by the dual notion that search priming acts by altering attention allocation, and that bistable perception depends on attention allocation (Brascamp & Blake, 2012; Dieter, Brascamp, Tadin, & Blake, 2016; Dieter & Tadin, 2011; Ling & Blake, 2012; Zhang, Jamison, Engel, He, & He, 2011). These two facts together suggested to us that search priming should be able to influence bistable perception, in spite of our inability to find such an influence in prior work. One example of evidence for attentional influences on bistable perception is that perception at the onset of an ambiguous stimulus is influenced by attentional cues (Chong, Tadin, & Blake, 2005; Chopin & Mamassian, 2010, 2011; Dieter, Melnick, & Tadin, 2016; Kristjánsson, 2009; Mitchell, Stoner, & Reynolds, 2004; Ooi & He, 1999). For example, Mitchell et al. (2004) and Chong and Blake (2006) showed that a transient attentional cue affects the interpretation of a subsequent ambiguous stimulus. The approach in both studies involved binocular rivalry in which incompatible images are presented to the left and the right eye, leading to two possible percepts. By precueing one of the two images by applying a movement or contrast increase, the cued image was predominantly perceived on subsequent presentations. Further evidence for a role of attention allocation in bistable perception includes the finding by Ooi and He (1999) that perception during binocular rivalry could be biased by presenting a pop out cue to one eye, such that the image presented to this cued eye preferentially gained perceptual dominance.

Further evidence suggests that attention allocation in visual search biases perceptual conflict toward the features of a preceding target. Chopin and Mammassian (2010) presented two arrays with contrasting orientations to the left and the right eyes. Observers performed a search task and a bistable perception task on alternating trials. On search trials, observers reported the location of the target that had a lower contrast than surrounding items, whereas on bistable trials observers reported the orientation of the dominant array, continuously for 12 seconds. When the orientation of the array that contained the search item became predictable, observers were more likely to report this orientation during the onset of bistable trials. The authors concluded that task relevance cued perception to favor the orientation that would improve search performance (note, however, that search performance was not measured). Similarly, in a subsequent study, Chopin and Mamassian (2011) found that the surface of an ambiguous stimulus was more often perceived in the front when this surface contained a search target. While their results did not show a relation between search and perceptual biases directly, they show a link between the processes that are involved in both search perception, and, importantly, that an attentional shift between stimulus features may affect both search and perceptual outcomes (see Kristjánsson, 2009 for converging results).

Given this indirect evidence that search priming, by altering attention allocation, may influence bistable perception, we considered whether the lack of such an influence in our previous work was due to an incidental experiment design choice. In that previous study (Brinkhuis et al., 2015), we presented, at different moments, an ambiguous stimulus at fixation or visual search stimuli at a fixed distance around fixation. While this spatial arrangement is in correspondence with prior work on sensory memory (typically studied at fixation) and on visual search priming (typically studied using extrafoveally presented items), this difference in spatial locations may have prevented any interaction between the two trial types. In particular, sensory memory for bistable perception is confined to a narrow spatial range (Chen & He, 2004; Knapen, Brascamp, Adams, & Graf, 2009). Similarly, attentional biasing of perception also falls off across space (Fischer & Whitney, 2014). Any effect of search priming on bistable perception may therefore be spatially restricted as well. Here, we therefore ask whether search priming affects bistable perception when search items and ambiguous images overlap retinotopically. As the facilitation of target selection relies on the repetition of target and distractor features, we compared the influence of target items and distractor items on subsequent ambiguous displays presented at the same position.

## Methods

### Participants

Eight observers (mean age = 30.75 years; standard deviation [*SD*] = 3.27)) participated. Seven of them, including the current first author, had experience with psychophysics tasks. Except for this author, participants were naive to the goal of the experiment. Participation was voluntary, and observers did not receive payment or study credits. The experiment was conducted in accordance with the Declaration of Helsinki.

### Apparatus

We used Python and Psychopy to create and present stimuli (Peirce, 2007) on a 1,920 by 1,200 pixels, 60-cm-wide thin-film transistor -display at 60 Hz, at a distance of approximately 60 cm from eye position. To ensure that participants kept a stable view and constant distance relative to the display throughout the experiment, head position was held constant with a chin rest.

### Stimuli

We presented animated rotating spheres by showing white (123.14 cd/m^2^) circular dots that were scattered across its surface, against a gray background (28.58 cd/m^2^). Specifically, 64 dots were positioned by creating 16 imaginary rings at equal distances along the sphere’s vertical axis, and on each ring drawing 4 dots, placed at random radial positions, but with equal distance to one another. The top, bottom, and center rings of the spheres were dot-free. The distance between each ring of four dots was 0.15° (visual angle). The spheres rotated around their vertical axis at a speed of 0.17 cycles per second.

**Figure 1.**
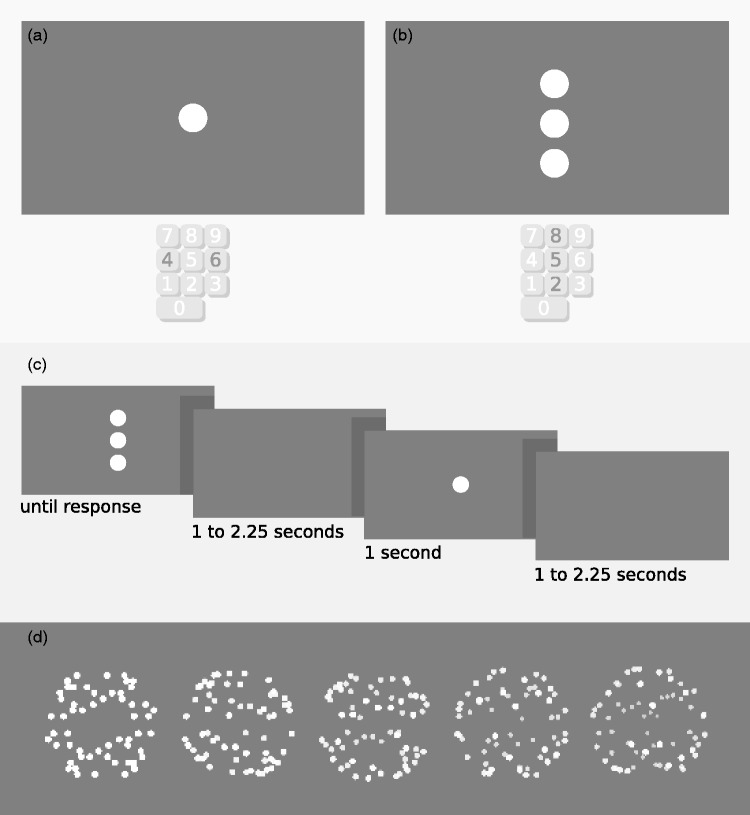
Experiment design. Panel (a) shows a simplified representation of the ambiguous display, and panel (b) shows the search display. The corresponding response buttons for each display are highlighted under both panels. The middle panel (c) shows the displays in sequence and their durations interleaved by blank displays that contained only a fixation dot. Finally, the lower panel (d) shows the five levels of disambiguation; where the left sphere was fully ambiguous and the right sphere was most strongly disambiguated through three gradually amplified depth cues: (i) decreasing dot size, (ii) reducing dot luminance, and (iii) enhancing perspective (note that, while differences in dot luminance and size can be appreciated in the figure, the full three-dimensional experience is not elicited without the dot motion that was present in our actual stimuli).

We presented two types of displays, as shown in Figure 1. One display involved the visual search paradigm. Three vertically aligned spheres were presented. The center sphere was presented at the center of the screen, the top sphere’s center was presented 3° above the central sphere, and the bottom sphere’s center was presented 3° below the central sphere. Depth cues were applied to the spheres by decreasing dot sizes as a function of dot depth position. Dot sizes ranged from 0.15° to a minimum of 0.03° for the farthest dots. In addition, dot luminance gradually decreased as a function of the dot’s depth position, to 84.0 cd/m^2^ at minimum. A third depth cue was added by linearly scaling horizontal and vertical coordinates as a function of the dot depth position such that the horizontal and vertical positions of the farthest dot were 20% closer to the central horizontal and vertical axes of the sphere relative to the dots closest to the observer, giving the impression of perspective and further disambiguating the sphere’s rotation direction, resulting in unambiguous leftward (i.e., clockwise when viewed from the top) or rightward rotation (i.e., counterclockwise when viewed from the top). After applying this perspective cue, the spheres were scaled such that their outline diameter remained 2.4°. One of the spheres always rotated in the opposite direction to the other two spheres and was the target of the visual search task. The rotation direction of the spheres was randomly set on each trial. On search display trials, participants were asked to respond by indicating the position of the oddly rotating sphere using the eight, five, and two keys on the numeric keypad of the keyboard, corresponding to the top, middle, and lower sphere, respectively.

The second display type involved the presentation of a single sphere with varying levels of ambiguity regarding rotation direction on each occurrence. The different levels of ambiguity were implemented by using different gain factors for the scaling of dot size, luminance, and dot placement as a function of distance. In particular, dot size decreased linearly as a function of dot depth position to 0.09°, 0.105°, 0.12°, 0.135°, or was constant for a fully ambiguous sphere. Dot luminance, in turn, faded to 73.01, 84.0, 96.0, 108.73 cd/m^2^, or remained constant (123.14 cd/m^2^). For the perspective cue, the horizontal and vertical positions of the dots farthest from the observer were scaled to be 40%, 30%, 20%, 10%, or 0% closer to the central horizontal and vertical axes relative to the dots that were closest to the observer. On these trials where a single sphere was presented, from here on referred to as *ambiguous trials*, participants were asked to respond by indicating perceived rotation direction using the four and six keys on the numeric keypad, corresponding to leftward and rightward rotation, respectively.

A central fixation dot was presented continuously throughout the experiment. The dot had a diameter of 0.15°. Because the central ring of each sphere did not contain dots, there was no overlap between moving dots and the central fixation dot, to ensure that it would not affect the percept of the central sphere.

### Procedure

Each experiment run consisted of a sequence of one, two, or three search trials followed by a single ambiguous trial. Each block contained 75 of those sequences or 225 trials in total. Search displays were presented for 2.5 seconds, or until a response was given, and ambiguous displays were presented for 1 second. Between each two trials, there was a period of 1 to 2.25 seconds where no stimulus was presented, except for the central fixation dot. The duration of each experiment block was about 12 minutes. Participants performed 4 blocks in each session and returned for three sessions for a total of 12 blocks.

To encourage participants to maintain eye fixation at screen center, we stressed that it was important to fixate on the central dot. Furthermore, we introduced a second task parallel to the main task. On random occasions, at the offset of either a search display or an ambiguous display, the fixation dot changed luminance from white to light gray (54.68 cd/m^2^) or the other way around. Participants were instructed to respond, using the space bar, each time the luminance of the fixation dot changed. Participants received points, added for each correct response, and subtracted for each incorrect response. Specifically, for each correctly identified luminance change, participants received 400 points, while for each missed change, 400 points were subtracted. Furthermore, during the search task, each correct response regarding the search target was rewarded with 200 points and for each incorrect response, 200 points were subtracted. During the ambiguous display, each response was rewarded with 100 points, regardless of what the response was. The score was displayed for 500 milliseconds after each response or at stimulus offset when the fixation dot luminance changed but was not reported. Furthermore, the score was presented at the position of the central fixation dot, in green for correct responses and in red for incorrect responses. Prior to the start of the first session, participants were asked to practice the task, which they continued until they reached 5,000 points; these trials were not used in the analysis.

### Analysis

Responses were recorded continuously throughout experiment runs. We selected each first response after the onset of search displays, ignoring response corrections. On ambiguous displays only the last response after stimulus onset (until the next stimulus onset) was selected, allowing participants to correct their response of perceived rotation direction. Note, however, that participants were not instructed that they could change their choice and consequently rarely did so.

Before conducting statistical analysis, we preprocessed the search data in the following way. First, we excluded outliers that were defined as (RTs more than three *SD*s above the mean RT or RTs lower than 500 milliseconds. We included all trials with incorrect response into our analyses. Next, we subtracted the linear slope of RTs, corresponding to a gradual slowing that might be associated with waning motivation, for each experiment block. We then normalized the data by taking the log of RTs to decrease distribution skew and by subtracting the mean RT and dividing by the *SD* for each experiment run, thus resulting in a detrended, *z*-scored logarithm of the RT. Finally, we rescaled the RTs to the mean *SD* across experiment runs and added the grand mean. Note that the last step scales the RTs so they reflect averages across subjects and that RTs at the individual level become less meaningful. Finally, we concatenated the data across all participants, and all experiment runs for further analysis.

To assess significance, we used the R statistical software package (R core team, 2017) and the lme4 library (Bates, Mächler, Bolker, & Walker, 2015). We fitted a linear mixed model to the concatenated search data to assess search priming using the lmer function in R. We fitted generalized linear mixed models to the perceptual choice data to assess the relation between search priming and bistable perception using the glmer function in R and a logistic link function. In all models, individual intercepts were modeled as random effects. The interpretation of ambiguous spheres was modeled as a binary dependent variable, where leftward and rightward responses were modeled as 0 and 1, respectively. We used an iterative approach to select the model that best fitted the data. All models were fitted using Laplace approximation (Bolker et al., 2009). By adding one factor of interest at a time and comparing the expanded model with the initial model using a likelihood ratio test, we identified significant predictors using a cutoff at a *p* value of .05. We calculated approximations of the Bayes factors (BFs) from the Bayesian Information Criteria of both models using the following Equation 1 derived from (Wagenmakers, See, & Cohen, 2007),

(1) $${\text{BF}}_{12}={\text{e}}^{\frac{{\text{BIC}}_{\text{M}1}-{\text{BIC}}_{\text{M}2}}{2}}$$

where BF_12_ is the BF of Models M_1_ and M_2_, M_1_ is a baseline model and M_2_ is the expanded model. Here, a BF value larger than 1 suggests the expanded model fits the data best, whereas a value lower than 1 favors the baseline model (Dienes, 2014).

## Results

**Figure 2.**
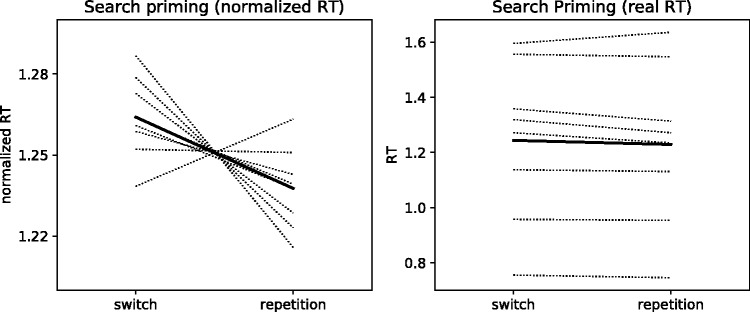
Normalized RTs (left) and real RTs (right) for each subject (thinner dotted lines) and averaged across subjects (thicker solid line), averaged over trials in which the target and distractor repeated and trials in which target and distractor rotation direction switched. RT = response time.

On average, participants responded correctly on 90% (*SD* = 7%) of the search trials. Figure 2 displays average RTs per observer, for search trials where target and distractor rotation directions repeated, and for search trials where they switched.

To assess whether search priming affected bistable perception, it is fundamentally important that our paradigm elicited search priming. We conducted a linear mixed-model analysis of the normalized search RTs, dependent on repetition of target-rotation direction across search displays. Specifically, we first fitted a baseline linear mixed model to the search data, including only the intercept. We then compared the baseline model with an expanded model that included a parameter for target rotation repetition (see Models A1 and A2 in Table 1). Estimation of the parameters showed a decrease in RTs when target rotation repeated. A likelihood ratio test yielded significantly improved performance of the expanded model over the baseline model, χ^2^(1) = 14.27, *p* = .0002, supported by a BF of 10.5, suggesting that RTs were indeed reliably faster when target rotation repeated.

**Table 1.** Model Overview.

Model	R-function	Description
A1	lmer	Normalized RT ∼ intercept
A2		Normalized RT ∼ intercept + target repetition
B1	glmer	*P*(→) ∼ intercept
B2		*P*(→) ∼ intercept + baseline
B3		*P*(→) ∼ intercept + baseline + AS prior response
B4		*P*(→) ∼ intercept + baseline + CS rotation
B5		*P*(→) ∼ intercept + baseline + CS rotation + CS role + (CS rotation * CS role)

*Note.* To assess search priming, we compared how well linear mixed models A1 and A2 fitted RTs. For the next part of our analyses, we compared generalized linear mixed models. First, we compared B2 and B3 to assess biased perceptual choice depending on the previous interpretation. In each Model B, we predict the probability that the observer responded with the right-arrow key; *P*(→). We compared Models B2 and B4 to assess the influence of the central search item on perceptual choice, and Models B4 and B5 to assess whether the role of the CS interacted with the influence of the CS on perceptual choice. All models included an intercept and models B2 to B5 included a regressor for nine different ambiguity levels, ranging from unambiguously rotating leftward, through fully ambiguous rotation, to unambiguously rotating rightward. AS = ambiguous sphere; CS = central sphere; RT = response time.

**Figure 3.**
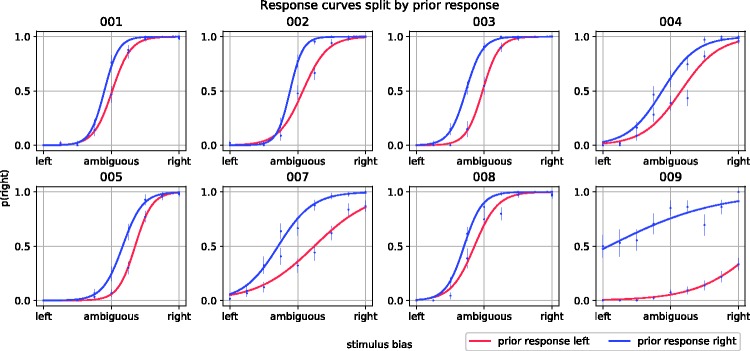
Probabilities that single spheres that interleaved search trials were perceived to rotate rightward. The red lines show perceptual bias when the previous sphere was perceived to rotate leftward, whereas blue lines show perceptual bias when the previous sphere was perceived to rotate leftward.

Another important prerequisite to testing our hypothesis is that perception of the ambiguous stimuli was susceptible to perceptual biases through trial history. This may not be the case if, for example, our method to disambiguate the spheres worked too well or when observers were already too strongly biased toward a certain interpretation at the start of the experiment. To rule out this possibility, we fitted a generalized linear mixed model, with a logistic link function, to test for influences of prior perception during the previous ambiguous trial on perception during the current ambiguous trial (i.e., influences of sensory memory; depicted in Figure 3). We first created a baseline model with the probability of a rightward response to an ambiguous display, indicating perceived rightward rotation, as the outcome variable. The model (Table 1; Model B2) included an intercept and a predictor that reflected the level of physical ambiguity of the ambiguous display. The ambiguity levels ranged from −1 to 1 in nine equally distanced steps, reflecting leftward rotation and rightward rotation, respectively, going through 0, reflecting a physically fully ambiguous sphere. These values mapped onto the steps we took to disambiguate the spheres as described in the Methods section. We then included a predictor for the response (left; 0, right; 1) on the previous ambiguous display (Table 1; model B3) and found that this model, following a likelihood ratio test, fitted the data significantly better than the baseline model, χ^2^(1) = 376.21, *p* < .0001, with a very high associated BF (5.81 × 1,079). These results show convincingly that observers’ perception was biased by trial history.

**Figure 4.**
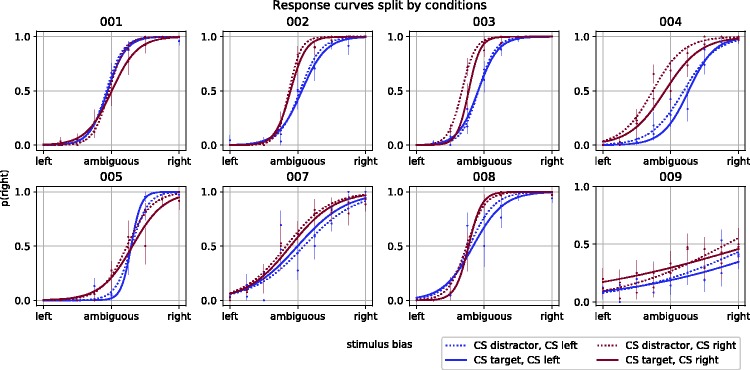
Probabilities of perceived rotation directions of the single spheres that interleaved search trials. The probability that the sphere was perceived as rotating rightward (on the *y*-axis) relied, generally, on the induced stimulus bias (*x*-axis). Furthermore, the red lines represent perceptual bias when the CS in the preceding search display was rotating leftward, whereas the blue lines show perceptual bias when the preceding CS was rotating rightward. Solid lines show perceptual bias when the CS was the target, whereas dotted lines show perceptual bias when the CS was a distractor. CS = central sphere.

After having, in this fashion, established the presence of trial history effects within each trial type (search and perception), we next investigated trial history effects from search trials onto trials with ambiguous displays: the main objective of the present work. To get one step closer to that objective, we iteratively expanded the previous model (see Tables 1 and 2 for an overview of all of these models) to assess the influence of target selection during search on perception during subsequent ambiguous displays, on top of the history effect that was evident for ambiguous displays. The response probabilities that were modeled in the next two analyses are depicted in Figure 4, as a function of the level of the graphically induced bias on ambiguous displays. The central sphere presented during search displays overlapped with the position of the sphere presented during the ambiguous display. As our objective was to search for retinotopically specific effects of search priming on bistable perception, we specifically assessed the influence of this central search item on bistable perception (i.e., on the probability the ambiguous spheres would be perceived to have a rightward rotation direction). To assess this influence, we first created a new model that included a predictor for the rotation direction of the central sphere on the search trial that immediately preceded an ambiguous trial (Table 1; model B4). This expanded model fitted the response data significantly better, χ^2^(1) = 70.36, *p* < .0001, supported by a large BF (2.24 × 1,013). The data therefore convincingly show that rotation direction of the overlapping sphere biased observers toward perceiving the same rotation direction of the overlapping subsequent ambiguous sphere.

**Table 2.** Iterative Model Comparisons Using Likelihood Ratio Tests.

Model	R-function	AIC	BIC	χ^2^	*df*	Pr(>χ^2^)
A1	lmer	14,143.50	14,166.18	NA	NA	NA
A2		14,131.23	14,161.47	14.27063	1	.0002
B1	glmer	9,681.45	9,695.21	NA	NA	NA
B2		4,981.92	5,002.56	4,701.53	1	.000
B3		4,607.71	4,635.24	376.21	1	.000
B4		4,539.35	4,573.75	70.36	1	.000
B5		4,539.93	4,588.10	3.42	2	.181

*Note.* Model A2 included an intercept and a predictor for target repetitions, whereas model A1 included only the intercept. Model A2 fitted the data better than Model A1. Each Model B includes an additional predictor for the response on ambiguous displays. Model B4 that included factors for the prior response on ambiguous trials and the central sphere’s direction on the immediate preceding search display fitted the data best, as indicated by the lower BIC. AIC = Akaike information criterion; BIC = Bayesian information criterion; NA = not applicable.

The above analysis, however, while showing a priming effect of the central sphere during the most recent search trial on perception during an ambiguous trial, does not address our central question: whether visual search priming influences perception of an ambiguous display. After all, this analysis does not take into account whether the central sphere was a target or a distractor during this most recent search trial. In other words, the analysis simply tests whether perception of an ambiguous stimulus is affected by prior presentation, at the same retinal location, of an unambiguous stimulus that resembles one of its interpretations, regardless of any role the unambiguous stimulus may have as part of a visual search array. Such sensory memory effects due to disambiguated input have been demonstrated before (Kanai, Knapen, van Ee, & Verstraten, 2007; Kanai & Verstraten, 2005; Long & Moran, 2007; Long, Toppino, & Mondin, 1992).

Our next analysis therefore takes the role of the central sphere during search (i.e., whether it is a target or distractor) into account, to address the question whether a target at the central location affects subsequent perception differently than a distractor at the central location does, as would be predicted in the case of retinally specific effects of search priming on perception. Specifically, we added a predictor to the model reflecting the interaction between the role and the direction of the central sphere (Table 1; Model B5). In other words, we added, to our earlier model that included the rotation direction of the preceding sphere as a predictor, a second predictor specifying whether the most recent central sphere was a target or a distractor. If history effects due to visual search also affect bistable perception, in a retinally specific fashion, then we expect that the rotation direction of the central sphere would have a particularly strong priming effect on bistable perception if the central sphere was the target, whereas the effect may be weaker if the central sphere was a distractor, or may even be reversed, analogous to the effect of role reversals in visual search (Chetverikov et al., 2016; Chetverikov, Campana, & Kristjánsson, 2017). The expanded model did, however, not fit the response data significantly better, χ^2^(2) = 3.42, *p* = .181, than the model that did not take the role of the central sphere into account. Indeed, a BF of 7.67 × 10^−4^ showed strong evidence in favor of the simpler model, thus suggesting that the search item’s role did not bias the perceived rotation direction of the ambiguous sphere. Together with the results of the previous analysis, this shows that effects of prior visual search on subsequent bistable perception in this experiment were restricted to retinally specific sensory memory effects (i.e., priming of the rotation direction of the central search item) that are unrelated to the search task itself (i.e., no influence of whether this item was a target or distractor).

To assess the consistency of these effects across observers, we reran the analyses for Models B1 to B5 (see Table 1) for each observer with session included as random factor. We found the same pattern of significant results across observer, except for Observer 1 and Observer 5. The above results corroborate our previous finding (Brinkhuis et al., 2015) that history effects in visual search and history effects in the perception of ambiguous displays are unrelated in the sense that the traces left by visual search do not influence bistable perception. The results also expand on them by showing that this even holds when retinal overlap between the search display element and the ambiguous stimulus is ensured.

**Figure 5.**
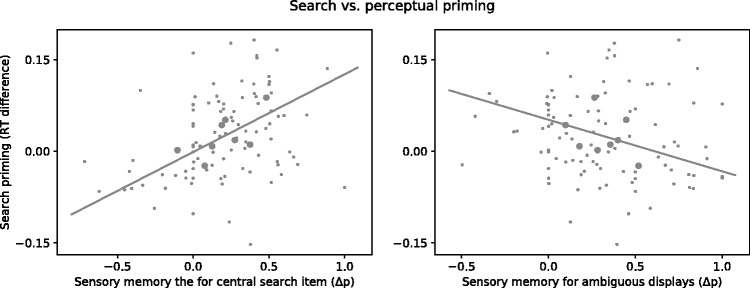
Search priming strength (*y*-axis) against sensory memory strength (*x*-axis) for central search items (left) and for ambiguous displays (right). In the left panel, the *x*-axis represents the difference between the probabilities (Δ*p*) of giving a right response when the preceding central sphere rotated leftward and the preceding central sphere rotated rightward. In the right panel, the *x*-axis represents the difference between the probabilities (Δ*p*) of giving a right response when the preceding response to the ambiguous display was left and when it was right. Smaller gray dots show the priming strengths across experiment runs (i.e., 12 blocks per observer), whereas bigger gray dots show average priming strengths per observer across blocks. The linear function was fitted to the average priming strengths across blocks. RT = response time.

The results described earlier provide a negative answer to the main question of the present work, that is, whether sensory memory and search priming are closely related phenomena so that search priming could also affect ambiguous figure perception. In an exploratory analysis, we next examined the possibility of a more indirect relation. In particular, our experiments elicited both search priming and sensory memory simultaneously, and we tested whether the strengths of the two types of history effects were correlated on an observer-to-observer or block-by-block basis. Such a correlation might be expected if some more general mechanism (e.g., arousal) affects both types of history effects similarly. We therefore modeled whether the search priming strengths per experiment block were predicted by the strength of sensory memory, including observers as a predictor of random effects (see Figure 5; left panel).

Specifically, we calculated the strength of search priming as the difference between mean normalized RTs on trials where the target and distractor repeated and on trials where they switched. This was done for each block and for each observer. We also calculated the strength of sensory memory for search items, by assessing the difference between the influence of leftward and rightward rotating central search items on the probability of perceiving ambiguous spheres as rightward rotating, again per block and observer. In a linear mixed model, with search priming strength as the outcome variable, we found that the strength of sensory memory predicted the strength of visual search priming significantly, relative to a model including only the intercept, χ^2^(1) = 6.98, *p* = .008, with a substantial BF of 3.34. To further explore this effect, we also calculated the correlation between the averages per observer of perceptual biases and priming strengths. Doing so, we aimed to identify whether the correlation depended on structural differences between observers, possibly reflecting that observers used different strategies. We indeed found the same positive trend, *r*(8) = .60, *p* = .07, although this correlation was not quite significant.

Interestingly, the result was specific to a comparison between search priming and sensory memory elicited by the central search item: It did not arise when we compared search priming to sensory memory elicited by ambiguous displays (i.e., to the effect of one ambiguous trial on the next). Specifically, we replaced the predictor that reflects the perceptual bias due to the central search item, with one that reflects sensory memory elicited by the prior ambiguous display (Figure 5; right panel), and did not obtain the same result. Instead, the strength of visual search priming per block was not significantly predicted by sensory memory for ambiguous displays per block,χ^2^(1) = 1.29, *p* = .26, with a BF of 0.20, and per observer, *r*(8) = − .34, *p* = .41. The relation between search priming and sensory memory was therefore specific to sensory memory elicited by search items.

## Discussion

While search priming effects spread across the visual field, the range at which bistable perception can be influenced by history effects has been found to be narrower (Chen & He, 2004; Knapen, Adams, & Graf, 2009). We measured interactions between retinotopically overlapping search items and ambiguous figures, to examine whether priming of visual search can influence perception of ambiguous stimuli. When search items and ambiguous figures were presented at fixation, bistable perception was indeed significantly biased by the prior search display, but this influence was not related to search priming. Instead, the perceptual bias induced by the search item presented at the central location did not rely on the role of this item (target or distractor). Rather, bistable perception was biased toward the percept that resembled this foveal search item regardless of its role. The current results confirm our earlier finding (Brinkhuis et al., 2015) that there is no effect of search priming, as such, on bistable perception and extend this finding by showing that this is even true when there is retinal correspondence between the bistable stimuli and search items used. Our results, therefore, further support the notion that sensory memory and search priming are independent phenomena and, by inference, that the resolution of perceptual ambiguity relies on distinct processes from competition between attended and ignored items during visual search.

In an exploratory analysis, we did find a more indirect link between search priming and sensory memory for ambiguous figures. Specifically, the strength of the sensory memory for search items predicted the strength of visual search priming. This suggests a weaker relation between the biasing effects of sensory memory and of search priming. For instance, both types of visual memory may rely on a common, nonspecific factor like arousal or the level of attention given to display items. In the present work, the correlation between the strengths of the two types of history effects reached significance when analyzed at the level of individual runs and showed a similar trend when analyzed across observers, instead of across experiment runs. Further study on this observer-to-observer relation should reveal whether this trend reflects a real effect. Such consistent differences may indicate differences in physiology or strategy between observers that specifically affect sensory memory for search items.

The current finding that the mere presence of a search item at the central location primes subsequent bistable perception, regardless of its role as either target or distractor, resembles findings in previous studies showing that perception can be biased by the mere viewing of prior unambiguous stimuli (Brascamp, Knapen, Kanai, van Ee, & van den Berg, 2007; Kanai et al., 2007; Kanai & Verstraten, 2005; Takeuchi, Tuladhar, & Yoshimoto, 2011). Similar to the present findings, those previous studies showed that an unambiguous stimulus can prime the corresponding interpretation so that it becomes dominant during subsequent viewing of a similar, ambiguous stimulus. Interestingly, these effects become more pronounced when attention is drawn toward stimulus characteristics corresponding to one of the two possible interpretations (Chong & Blake, 2006; Mitchell et al., 2004). The current lack of an interaction between search priming and bistable perception may therefore be interpreted in two ways. First, reorientation of attention toward the target may prime subsequent search but not bistable perception—the two tasks may rely on separate mechanisms. Second, it may be that observers did not reorient attention toward the target during a search but attended target and distractors to an equal extent. In that case, the search priming effect in this study, and in similar studies, may have relied primarily on the enhanced discriminability of target and distractor.

Analogously to the complementary effects of target and distractor items in visual search, perception is locally attracted toward previously attended stimuli or repelled from previously unattended stimuli (Fischer & Whitney, 2014; Fritsche, Mostert, & de Lange, 2017). While Whitney and Fischer found that perception is attracted to previously displayed stimuli when an observer is asked to report a certain orientation of a stimulus, Fritsche and others replicated this finding but found that perceptual judgment tasks yielded different results. Importantly, their results for different types of experiments relied on the same stimuli but were opposite in nature. The results specifically indicated that different biases are represented in different types of responses. For example, as suggested by Fritsche and others, a positively biased perceptual decision may rely on the previous response to a stimulus as well as on the perception of a preceding stimulus (Cicchini, Mikellidou, & Burr, 2017), whereas in parallel, perceptual judgments may rely on adaptation to that same stimulus. In other words, the effects of visual search on perception may depend on the method used to probe perception, suggesting that the perceptual signature of search priming may change when using a different paradigm. Importantly, though, here, we find no evidence that, specifically, the perception of ambiguous stimuli and target selection during search relies on shared mechanisms.

To summarize, we previously found that distinct effects of attentional and perceptual selection history did not interact. Here, we showed that the absence of those interactions was not due to a spatially narrow susceptibility of bistable perception for bias through prior visual search: When ensuring retinal overlap between search items and ambiguous stimuli, we found that search priming still does not alter perception of subsequent ambiguous stimuli. Instead, items in the search array can cause sensory memory irrespective of their role in search—an effect that has also been observed when no search is involved at all, and that is distinct from search priming. In an exploratory analysis, we found that the strength of this sensory memory was related to the strength of visual search priming, possibly reflecting that observers performed the search task with varied attention to search items across experiment blocks.

## References

[bibr1-2041669518812485] Ásgeirsson Á. G. Kristjánsson Á. Bundesen C. (2014) Independent priming of location and color in identification of briefly presented letters. Attention, Perception & Psychophysics 76: 40–48. doi:10.3758/s13414-013-0546-6. 10.3758/s13414-013-0546-6 24092356

[bibr2-2041669518812485] Bates D. Mächler M. Bolker B. Walker S. (2015) Fitting linear mixed-effects models using lme4. Journal of Statistical Software 67: 1–48. doi:10.18637/jss.v067.i01.

[bibr3-2041669518812485] Bolker B. M. Brooks M. E. Clark C. J. Geange S. W. Poulsen J. R. Stevens M. H. H. White J. S. S. (2009) Generalized linear mixed models: A practical guide for ecology and evolution. Trends in Ecology and Evolution 24: 127–135. doi:10.1016/j.tree.2008.10.008. 19185386 10.1016/j.tree.2008.10.008

[bibr4-2041669518812485] Brascamp J. W. Blake R. (2012) Inattention abolishes binocular rivalry: Perceptual evidence. Psychological Science 23: 1159–1167. doi:10.1177/0956797612440100. 22933458 10.1177/0956797612440100

[bibr5-2041669518812485] Brascamp J. W. Knapen T. H. J. Kanai R. van Ee R. van den Berg A. V. (2007) Flash suppression and flash facilitation in binocular rivalry. Journal of Vision 7: 1–12. doi:10.1167/7.12.12.Introduction. 10.1167/7.12.12 17997654

[bibr6-2041669518812485] Brinkhuis, M. A. B., Kristjánsson, Á., & Brascamp, J. W. (2015). Evidence for distinct mechanisms underlying attentional priming and sensory memory for bistable perception. *Journal of Vision*, *15*, 1–15. doi:10.1167/15.11.8. 10.1167/15.11.8 26270190

[bibr7-2041669518812485] Chen X. He S. (2004) Local factors determine the stabilization of monocular ambiguous and binocular rivalry stimuli. Current Biology 14: 1013–1017. doi:10.1016/j.cub.2004.05.042. 15182676 10.1016/j.cub.2004.05.042

[bibr8-2041669518812485] Chetverikov A. Campana G. Kristjánsson Á. (2016) Building ensemble representations: How the shape of preceding distractor distributions affects visual search. Cognition 153: 196–210. doi:10.1016/j.cognition.2016.04.018. 27232163 10.1016/j.cognition.2016.04.018

[bibr9-2041669518812485] Chetverikov A. Campana G. Kristjánsson Á. (2017) Representing color ensembles. Psychological Science 28: 1510–1517. doi:10.1177/0956797617713787. 28862923 10.1177/0956797617713787

[bibr110-2041669518812485] Chong, S. C., & Blake, R. (2006). Exogenous attention and endogenous attention influence initial dominance in binocular rivalry. *Vision Research*, *46*, 1794–1803. doi:10.1016/j.visres.2005.10.031. 10.1016/j.visres.2005.10.031 16368126

[bibr10-2041669518812485] Chong S. C. Tadin D. Blake R. (2005) Endogenous attention prolongs dominance durations in binocular rivalry. Journal of Vision 5: 1004–1012. doi:10.1167/5.11.6. 16441198 10.1167/5.11.6

[bibr11-2041669518812485] Chopin A. Mamassian P. (2010) Task usefulness affects perception of rivalrous images. Psychological Science 21: 1886–1893. doi:10.1177/0956797610389190. 21106886 10.1177/0956797610389190

[bibr12-2041669518812485] Chopin A. Mamassian P. (2011) Usefulness influences visual appearance in motion transparency depth rivalry. Journal of Vision 11: 18, doi:10.1167/11.7.18. 10.1167/11.7.18 21705461

[bibr13-2041669518812485] Cicchini G. M. Mikellidou K. Burr D. (2017) Serial dependencies act directly on perception. Journal of Vision 17: 6, doi:10.1167/17.14.6. 10.1167/17.14.6 29209696

[bibr14-2041669518812485] de Jong M. C. Knapen T. van Ee R. (2012) Opposite influence of perceptual memory on initial and prolonged perception of sensory ambiguity. PLoS One 7: e30595, doi:10.1371/journal.pone.0030595. 22295095 10.1371/journal.pone.0030595 PMC3266287

[bibr15-2041669518812485] Dienes Z. (2014) Using Bayes to get the most out of non-significant results. Frontiers in Psychology 5: 1–17. doi:10.3389/fpsyg.2014.00781. 25120503 10.3389/fpsyg.2014.00781 PMC4114196

[bibr16-2041669518812485] Dieter K. C. Brascamp J. Tadin D. Blake R. (2016) Does visual attention drive the dynamics of bistable perception? Attention, Perception, & Psychophysics 78: 1861–1873. doi:10.3758/s13414-016-1143-2. 10.3758/s13414-016-1143-2 PMC5014653 27230785

[bibr17-2041669518812485] Dieter K. C. Melnick M. D. Tadin D. (2016) Perceptual training profoundly alters binocular rivalry through both sensory and attentional enhancements. Proceedings of the National Academy of Sciences 113: 201602722, doi:10.1073/pnas.1602722113. 10.1073/pnas.1602722113 PMC5111677 27791061

[bibr18-2041669518812485] Dieter K. C. Tadin D. (2011) Understanding attentional modulation of binocular rivalry: A framework based on biased competition. Frontiers in Human Neuroscience 5: 1–12. doi:10.3389/fnhum.2011.00155. 22144958 10.3389/fnhum.2011.00155 PMC3228993

[bibr19-2041669518812485] Fischer J. Whitney D. (2014) Serial dependence in visual perception. Nature Neuroscience 17: 738–743. doi:10.1038/nn.3689. 24686785 10.1038/nn.3689 PMC4012025

[bibr20-2041669518812485] Fritsche M. Mostert P. de Lange F. P. (2017) Opposite effects of recent history on perception and decision. Current Biology 27: 590–595. doi:10.1016/j.cub.2017.01.006. 28162897 10.1016/j.cub.2017.01.006

[bibr21-2041669518812485] Kanai R. Knapen T. H. J. van Ee R. Verstraten F. A. J. (2007) Disruption of implicit perceptual memory by intervening neutral stimuli. Vision Research 47: 2675–2683. doi:10.1016/j.visres.2007.06.016. 17697690 10.1016/j.visres.2007.06.016

[bibr22-2041669518812485] Kanai R. Verstraten F. A. (2005) Perceptual manifestations of fast neural plasticity: Motion priming, rapid motion aftereffect and perceptual sensitization. Vision Research 45: 3109–3116. doi:10.1016/j.visres.2005.05.014. 16023173 10.1016/j.visres.2005.05.014

[bibr24-2041669518812485] Knapen, T., Brascamp, J., Adams, W. J., & Graf, E. W. (2009). The spatial scale of perceptual memory in ambiguous figure perception. *Journal of Vision*, *9*, 16–16. doi:10.1167/9.13.16. 10.1167/9.13.16 20055549

[bibr25-2041669518812485] Kristjánsson Á. (2009) Learning in shifts of transient attention improves recognition of parts of ambiguous figure-ground displays. Journal of Vision 9: 1–11. doi:10.1167/9.4.21.Introduction. 10.1167/9.4.21 19757930

[bibr26-2041669518812485] Kristjánsson Á. Driver J. (2008) Priming in visual search: Separating the effects of target repetition, distractor repetition and role-reversal. Vision Research 48: 1217–1232. doi:10.1016/j.visres.2008.02.007. 18374961 10.1016/j.visres.2008.02.007

[bibr27-2041669518812485] Lamy D. Yashar A. Ruderman L. (2013) Orientation search is mediated by distractor suppression: Evidence from priming of pop-out. Vision Research 81: 29–35. doi:10.1016/j.visres.2013.01.008. 23380440 10.1016/j.visres.2013.01.008

[bibr28-2041669518812485] Leopold D. A. Wilke M. Maier A. Logothetis N. K. (2002) Stable perception of visually ambiguous patterns. Nature Neuroscience 5: 605–609. doi:10.1038/nn851. 11992115 10.1038/nn0602-851

[bibr29-2041669518812485] Ling S. Blake R. (2012) Normalization regulates competition for visual awareness. Neuron 75: 531–540. doi:10.1016/j.neuron.2012.05.032. 22884335 10.1016/j.neuron.2012.05.032 PMC3419498

[bibr30-2041669518812485] Long G. M. Moran C. J. (2007) How to keep a reversible figure from reversing: Teasing out top-down and bottom-up processes. Perception 36: 431–445. doi:10.1068/p5630. 17455757 10.1068/p5630

[bibr31-2041669518812485] Long G. M. Toppino T. C. Mondin G. W. (1992) Prime time: Fatigue and set effects in the perception of reversible figures. Perception & Psychophysics 52: 609–616. doi:10.3758/BF03211697. 1287566 10.3758/bf03211697

[bibr32-2041669518812485] Maier A. Wilke M. Logothetis N. K. N. N. K. Leopold D. A. (2003) Perception of temporally interleaved ambiguous patterns. Current Biology 13: 1076–1085. doi:10.1016/S. 12842006 10.1016/s0960-9822(03)00414-7

[bibr33-2041669518812485] Maljkovic V. Nakayama K. (1994) Priming of pop-out: I. Role of features. Memory & Cognition 22: 657–672. Retrieved from http://link.springer.com/article/10.3758/BF03209251. 7808275 10.3758/bf03209251

[bibr34-2041669518812485] Mitchell J. F. Stoner G. R. Reynolds J. H. (2004) Object-based attention determines dominance in binocular rivalry. Nature 429: 410–413. doi:10.1038/nature02584. 15164062 10.1038/nature02584

[bibr35-2041669518812485] Ooi T. L. He Z. J. (1999) Binocular rivalry and visual awareness: The role of attention. Perception 28: 551–574. doi:10.1068/p2923. 10664754 10.1068/p2923

[bibr36-2041669518812485] Pastukhov A. (2016) Perception and the strongest sensory memory trace of multi-stable displays both form shortly after the stimulus onset. Attention, Perception, & Psychophysics 78: 674–684. doi:10.3758/s13414-015-1004-4. 10.3758/s13414-015-1004-4 26542402

[bibr37-2041669518812485] Pastukhov A. Braun J. (2008) A short-term memory of multi-stable perception. Journal of Vision 8: 7.1–7.14 doi:10.1167/8.13.7. 10.1167/8.13.7 19146337

[bibr38-2041669518812485] Pastukhov A. García-Rodríguez P. E. Haenicke J. Guillamon A. Deco G. Braun J. (2013) Multi-stable perception balances stability and sensitivity. Frontiers in Computational Neuroscience 7: 17, doi:10.3389/fncom.2013.00017. 23518509 10.3389/fncom.2013.00017 PMC3602966

[bibr39-2041669518812485] Pearson J. Brascamp J. (2008) Sensory memory for ambiguous vision. Trends in Cognitive Sciences 12: 334–341. doi:10.1016/j.tics.2008.05.006. 18684661 10.1016/j.tics.2008.05.006

[bibr40-2041669518812485] Peirce J. W. (2007) PsychoPy—Psychophysics software in Python. Journal of Neuroscience Methods 162: 8–13. doi:10.1016/j.jneumeth.2006.11.017. 17254636 10.1016/j.jneumeth.2006.11.017 PMC2018741

[bibr411-2041669518812485] R Core Team (2017). R: A language and environment for statistical computing. Vienna, Austria: R Foundation for Statistical Computing. http://www.R-project.org/.

[bibr41-2041669518812485] Sigurdardottir H. H. M. Kristjánsson Á. Driver J. (2008) Repetition streaks increase perceptual sensitivity in visual search of brief displays. Visual Cognition 16: 643–658. doi:10.1080/13506280701218364. 19325897 10.1080/13506280701218364 PMC2660840

[bibr42-2041669518812485] Takeuchi T. Tuladhar A. Yoshimoto S. (2011) The effect of retinal illuminance on visual motion priming. Vision Research 51: 1137–1145. doi:10.1016/j.visres.2011.03.002. 21396394 10.1016/j.visres.2011.03.002

[bibr43-2041669518812485] Tipper S. P. (1985) The negative priming effect: Inhibitory priming by ignored objects. The Quarterly Journal of Experimental Psychology 37: 571–590. doi:10.1080/14640748508400920. 4081101 10.1080/14640748508400920

[bibr44-2041669518812485] Treisman A. Gelade G. (1980) A feature-integration theory of attention. Cognitive Psychology 136: 97–136. Retrieved from http://www.sciencedirect.com/science/article/pii/0010028580900055. 10.1016/0010-0285(80)90005-5 7351125

[bibr45-2041669518812485] Wagenmakers E. J. See E. G. Cohen M. D. (2007) A practical solution to the pervasive problems of *p* values. Psychonomic Bulletin and Review 14: 779–804. doi:10.3758/BF03194105. 18087943 10.3758/bf03194105

[bibr46-2041669518812485] Zhang P. Jamison K. Engel S. He B. He S. (2011) Binocular rivalry requires visual attention. Neuron 71: 362–369. doi:10.1016/j.neuron.2011.05.035. 21791293 10.1016/j.neuron.2011.05.035 PMC3175243

